# Clinical Presentation, Diagnosis, and Management of Abdominal Tuberculosis in Pediatric Population: A Prospective Descriptive Study From a Tertiary Care Centre in North India

**DOI:** 10.7759/cureus.90491

**Published:** 2025-08-19

**Authors:** Enono Yhoshu, Deepak K Garnaik, Soumya Kashiv, Nowneet Bhat, Rajat Piplani, Amber Prasad, Intezar Ahmed, Satya Sree Balija

**Affiliations:** 1 Pediatric Surgery, All India Institute of Medical Sciences, Rishikesh, Rishikesh, IND; 2 Pediatric Medicine, All India Institute of Medical Sciences, Rishikesh, Rishikesh, IND; 3 Microbiology, All India Institute of Medical Sciences, Rishikesh, Rishikesh, IND

**Keywords:** abdominal tuberculosis, cbnaat, children, intestinal, koch’s abdomen, mycobacterium tuberculosis

## Abstract

Aims

Abdominal tuberculosis (TB) continues to be a common and challenging abdominal disease in children, with a nonspecific clinical presentation and poor outcomes in delayed diagnosis and complicated cases. We aimed to prospectively evaluate the clinical features and diagnostic workup of children with suspected abdominal TB, with a focus on early initiation of medical and surgical treatment and monitoring of therapeutic outcomes.

Materials and methods

A time-bound prospective observational study of all patients ≤ 17 years requiring admission with symptoms suggestive of abdominal TB from February 2020 to May 2023 was conducted. All necessary routine blood tests and imaging were done, and endoscopies and surgeries as needed by the patient were carried out. The data of the patients who were diagnosed as abdominal TB - probable or definitive - were analyzed.

Results

Forty-seven patients were recruited with a suspected diagnosis of abdominal TB. Thirty-four patients (24 females and 10 males) were diagnosed as abdominal TB - definite in 18/34 patients (52.94%) and probable in 16/34 patients (47.06%). The mean age was 12.20 ± 3.82 years (4-17 years). Median duration of the symptoms was three months (IQR = 1-5 months). The commonest symptoms were abdominal pain (94.11%), fever (73.52%), and loss of appetite and weight (70.58%). Twelve patients (35.29%) gave a positive history of contact with TB. There were 13/34 (38.23%) patients who had concomitant pulmonary and abdominal TB, and 21/34 (61.76%) patients who had only abdominal TB. Mantoux tuberculin skin test was performed in 20 patients, of which 9/20 (45%) were positive. A Cartridge-Based Nucleic Acid Amplification Test (CBNAAT) was performed in 30/34 patients (88.23%) from fluid or tissue samples, of which 11 patients (35.48%) showed CBNAAT positivity. Fifteen patients (44.11%) underwent surgery, 12 for intestinal perforation and three for intestinal obstruction. Out of the 18 definite cases of abdominal TB, 11/18 (61.111%) were CBNAAT positive, and 10/18 (55.55%) had histopathology suggestive of TB. The 16 probable cases of abdominal TB had a strong history and imaging suggestive of TB abdomen. A total of seven patients who underwent surgery for intestinal perforation expired. One patient developed a relapse, and four patients developed drug-induced liver injury (DILI).

Conclusion

Abdominal TB remains a common cause of acute abdomen in the pediatric population, often presenting with non-specific features and lacking a definitive diagnostic modality. Early detection through recognition of common clinical features, guided imaging, and timely sampling for confirmation is vital for initiating antitubercular therapy (ATT) and improving outcomes in abdominal TB.

## Introduction

India is one of the endemic areas of tuberculosis (TB) of various organ systems, accounting for around 26% of the global TB cases, preceding China and South Africa [1]. Abdominal TB is the seventh most common extrapulmonary TB site, after other more common sites - lymphatic, pleural, skeletal, genitourinary, miliary, and CNS forms. It comprises 3% of the total TB cases [2]. About 15-25% of abdominal TB cases have pulmonary TB concomitantly [3,4].

The clinical presentation of abdominal TB is nonspecific and has a wide spectrum, ranging from mild abdominal pain with lymphadenopathy and ascites to severe complications like intestinal perforation, bleeding, and obstruction. The diagnosis of abdominal TB has been another challenge, and a high index of suspicion, especially in an endemic area, is required. Histopathological observation of typical caseating granulomas has been reported in around 40% of abdominal TB, but more so in lymph node (LN) specimens only [5,6]. Isolation of acid-fast bacilli (AFB) by microscopy or culture in body fluids or tissue biopsies is the diagnostic gold standard, but the yield is low [7]. Body fluid adenosine deaminase (ADA) levels have a sensitivity and specificity of 93% and 96%, but they can have false negatives in malignant ascites and HIV [8]. The Cartridge-Based Nucleic Acid Amplification Test (CBNAAT) is a real-time PCR assay designed to detect *Mycobacterium tuberculosis* and rifampicin resistance. It offers rapid results with high specificity, though its sensitivity can be variable [9]. Radiological imaging may reveal suggestive but non-pathognomonic findings. The search for rapid clinical and investigational diagnosis and management of abdominal TB, which is crucial but still a challenge, continues.

Being an endemic country, we wanted to do a prospective observational analysis of all suspected abdominal TB patients, describing their varied patterns of presentation and diagnostic investigations, and following the outcomes with regard to initiation of medications or surgical intervention.

## Materials and methods

Study design and ethics statement

This prospective observational study was conducted in the Departments of Pediatric Surgery and Pediatric Medicine at the All India Institute of Medical Sciences, Rishikesh, over three years after getting approval from the Research Advisory Committee (RAC) and the Institutional Ethics Committee (IEC) (no. AIIMS/IEC/20/124).

Study population

All the patients ≤ 17 years of age who came to the Outpatient Department (OPD) of Pediatric Surgery and Pediatric Medicine or the Emergency with clinical features suspicious of acute, subacute or chronic abdominal TB, requiring admission were included in the study - abdominal pain, fever, loss of appetite or weight, vomiting, constipation, or diarrhea.

Study duration

A time-bound study from February 2020 to May 2023 was conducted.

Sample size

This was a time-bound study with convenience sampling. A total of 47 patients were initially recruited for this study with a suspected diagnosis of abdominal TB. Thirty-four patients were diagnosed with abdominal TB, and they were analyzed prospectively.

Study objectives

Primary Objective

The primary objective was to assess whether patients achieved clinical cure or experienced relapse following treatment for abdominal TB.

Secondary Objectives

The secondary objectives were to monitor mortality, therapeutic failure, treatment default, and poor adherence to therapy. 

Safety Objective

The safety objective was to document ATT-related adverse effects, particularly those that were serious, required hospitalization, or led to treatment changes.

Diagnostic workup

Initial evaluation of all the patients on admission included the following tests, which are routinely done and decided by the treating doctor: hemogram with erythrocyte sedimentation rate (ESR), Mantoux tuberculin skin test, renal function test (RFT), and liver function test (LFT), chest X-ray (CXR), and abdominal X-rays (AXR), ultrasonography (USG) abdomen, sputum/gastric aspirate acid-fast bacilli (AFB) staining and CBNAAT - GeneXpert® 4-module system (Cepheid, Sunnyvale, CA). If pleural or ascitic fluid were available, fluid serum ascites albumin gradient (SAAG), ADA, culture, and CBNAAT were done. For the patients who were not diagnosed by the above tests or for whom the decision of management needed further investigation, the following were done at the discretion of the treating doctors: contrast-enhanced computed tomography (CECT) abdomen and upper or lower gastrointestinal endoscopy with biopsy.

Management

Those who required surgical intervention for complications - intestinal obstruction, perforation, or bleeding - were managed according to surgical protocols. All attempts to obtain tissue for microbiological and histological diagnosis by radiologically imaging-guided, endoscopic, laparoscopic, or surgical exploration were made in all patients as and when feasible.

During the course of hospital stay, patients were categorized into two diagnostic groups of abdominal TB: definite and probable abdominal TB [10].

Definite abdominal TB included patients with microbiologically or histologically confirmed evidence of Mycobacterium tuberculosis. Microbiological confirmation was established either by a positive CBNAAT or by the demonstration of AFB on staining or culture from fluid or biopsy specimens. Histological confirmation was based on the presence of caseating granulomas on tissue biopsy.

On the other hand, probable abdominal TB was diagnosed in patients with a strong clinical suspicion of abdominal TB, where other differential diagnoses were excluded. These patients exhibited supportive findings on biochemical investigations, endoscopy, histopathology (such as non-caseating granulomas), radiological imaging, and intraoperative observations. A subjective or objective response to antitubercular therapy (ATT) further supported the diagnosis in these cases.

Various findings on imaging suggestive of TB abdomen are as follows: (a) gastrointestinal: bowel wall thickening, ulceration, narrowing; most commonly seen involving the ileocecal junction (ICJ); (b) peritoneal: ascites; omental and mesenteric thickening; omental and mesenteric masses and nodules; (c) lymph nodal: mesenteric, peripancreatic, periportal, paraaortic-nodal enlargement with central necrosis; nodal conglomeration forming masses; calcification in healed stage; and (d) visceral: organomegaly; micronodules; macronodules; and solitary lesion mimicking malignancy.

All definite and probable cases of abdominal TB were enrolled after obtaining informed consent and were prospectively followed during their hospital stay and after discharge. Patients diagnosed with multidrug-resistant (MDR) TB or initiated on MDR TB treatment were excluded from the study.

ATT, as per the Directly Observed Treatment Short Course (DOTS), was started for patients diagnosed as one of the above categories of abdominal TB. ATT included four drugs, which were weight-based. The intensive phase for the initial two months consists of Isoniazid (H), Rifampicin (R), Pyrazinamide (Z), and Ethambutol (E), followed by a maintenance phase of two drugs (HR) for four months. Operated patients who were unable to start oral ATT initially were started on second-line ATT, which was later switched to oral ATT once they were able to tolerate it.

Follow-up

The patients’ responses to treatment were assessed and included at least two follow-up visits in the outpatient department - (a) at two months of initiation of ATT and (b) at the end of the continuation phase.

In Pediatric Medicine OPD, the following were looked at: general condition, weight gain, and getting back to their daily routine, LFT, and other tests as per the status of the patient. In the Pediatric Surgery OPD, recurrence of abdominal symptoms and other tests were assessed as per the status of the patient.

**Figure 1:**
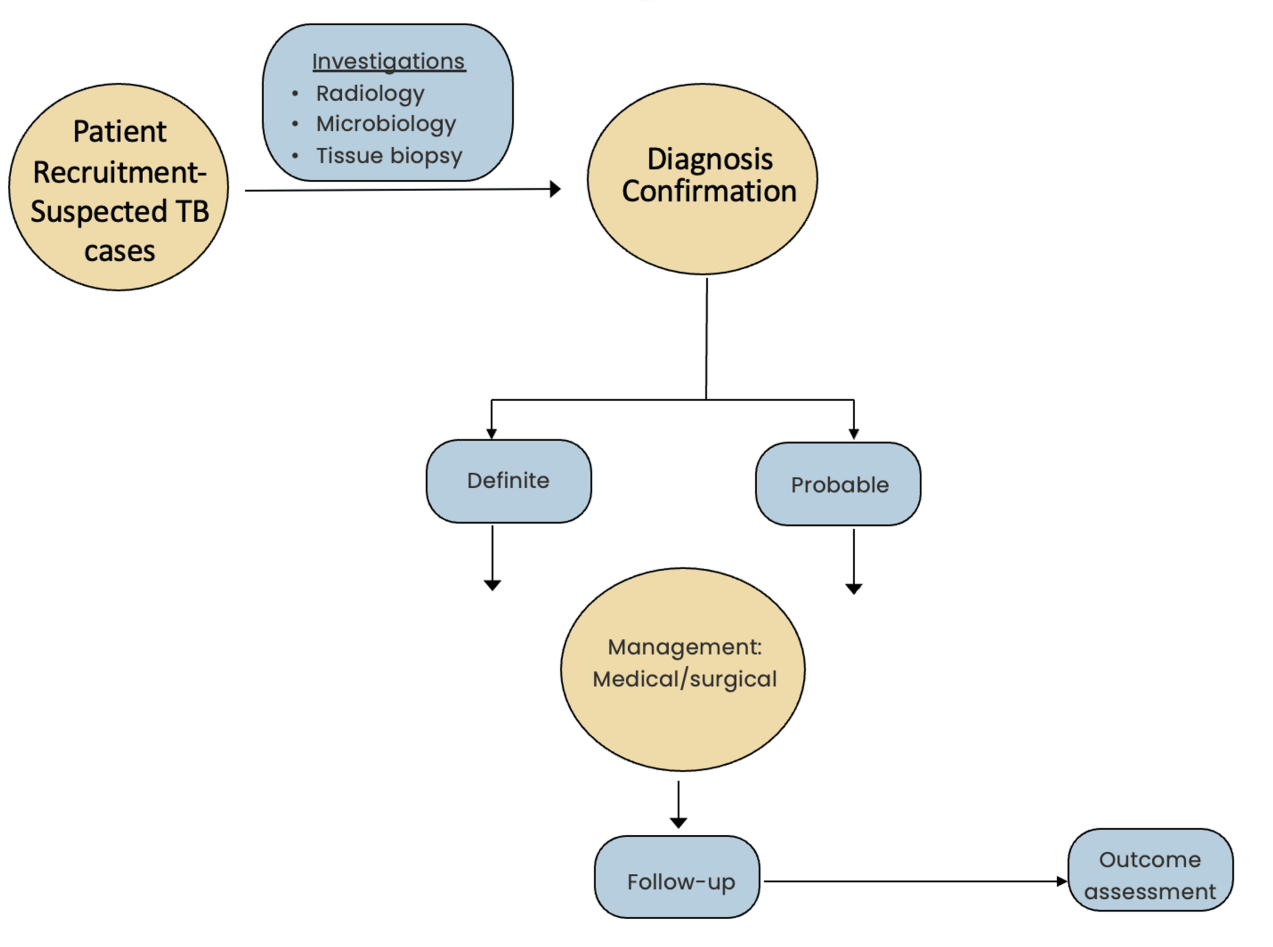
Flow chart of the study design

Figure 1 depicts the flow chart of the study design.

Statistical analysis

Data analysis was done using descriptive statistics, with all analyses performed using Microsoft Excel (Microsoft® Corp., Redmond, WA). Categorical variables such as gender, type of abdominal TB (definite or probable), symptom prevalence, diagnostic test outcomes (Mantoux test, CBNAAT), and surgical interventions were summarized using frequencies (n) and percentages (%). Continuous variables, including age, duration of symptoms, hemoglobin levels, and serum-ascites albumin gradient (SAAG) values, were described using mean and standard deviations (SD), median, and interquartile range (IQR).

## Results

Forty-seven patients with a provisional diagnosis of abdominal TB were recruited. Upon further evaluation, 34 were confirmed as having abdominal TB, with 18 (52.94%) meeting criteria for a definite diagnosis and 16 (47.06%) classified as probable cases. These 34 patients were included in the study. The remaining 13 were subsequently diagnosed with other conditions during their hospital stay: five with inflammatory bowel disease, three with lymphoma, two with pelvic inflammatory disease, and three with non-specific acute abdomen.

The mean age of the patients was 12.20 ± 3.82 years (four to 17 years), which included 10 males and 24 females. Median duration of the symptoms that the patients suffered from at the time of presentation to the institute was three months (IQR = 1-5 months). The clinical presentation of our patient population was as follows (Table 1). 

**Table 1:** Details of the clinical presentation of the patient population *qSOFA - Quick Sequential Organ Failure Assessment Score The presence of at least two of the following criteria strongly predicts the likelihood of poor outcome in out-of-ICU patients with clinical suspicion of sepsis: (1) RR ≥ 22/min; (2) altered mentation (GCS < 15); and (3) SBP ≤ 100 mmHg.

Parameters	Number of patients n/34 (%)
Symptoms	
Abdominal pain	32 (94.11%)
Fever	25 (73.52%)
Loss of appetite and weight	24 (70.58%)
Vomiting-bilious/non-bilious	19 (55.88%)
Abdominal distension	15 (44.11%)
Diarrhea	6 (17.64%)
Constipation	7 (20.58%)
Cough - with/without sputum	4 (11.76%)
Signs	
Sick at presentation (qSOFA >2)*	17 (50%)
Weight	
Normal (±2 SD)	13 (38.23%)
Underweight (-2 SD to -3 SD)	3 (8.82%)
Severely underweight (< -3 SD)	18 (52.94%)
Pallor	12 (35.29%)
Icterus	2 (5.88%)
Peripheral lymphadenopathy	2 (5.88%)
Chest findings (crepitations and/or decrease air entry)	11 (32.35%)
Abdominal distension ± ascites	21 (61.76%)
Guarding ± rigidity	10 (29.41%)

Twenty-two patients (64.70%) received treatment from other hospitals for their symptoms before presentation to our center. Twelve (35.29%) patients gave a positive history of contact with TB patients in their family or other associates. There were 13/34 (38.23%) patients who had concomitant pulmonary and abdominal TB, and 21/34 (61.76%) patients who had only abdominal TB.

The mean hemoglobin level was 10.11 ± 2.14 g/dL (range 4.8-13.75). Mantoux tuberculin skin test could be done in 20 patients, of which 9/20 (45%) were positive and the rest were negative. A total of 24 patients had fluid in the abdomen; out of these, 11 were perforated and needed surgical exploration. Two patients had mild ascites, which could not be aspirated for analysis; one had a rectovesical pouch collection, which showed pus on aspiration and was positive for CBNAAT. The mean SAAG value for the rest of the patients was 0.66 ± 0.19 (range 0.3-0.9), which was suggestive of exudate. Aerobic cultures were performed in 17 patients, of which five yielded positive results - *Klebsiella* in one case and *Escherichia col*i in four cases.

CBNAAT was performed in 30/34 patients (88.23%) from fluid (ascitic or sputum or gastric aspirate or intraoperative peritoneal or percutaneous abdominal pus), or from tissues (endoscopic intestinal biopsies or LNs or inflamed omentum or bowel perforation edges). Four patients did not have any pleural or peritoneal fluid to be sampled for CBNAAT, nor did they need a nasogastric tube for management (to take a gastric aspirate for CBNAAT), though they had features suggestive of TB abdomen on imaging. They had mesenteric lymphadenopathy, but we did not sample them. Eleven patients (36.66%) showed CBNAAT positivity from different samples (Table 2).

**Table 2:** CBNAAT sample fluid/tissue and reports from the patient population CBNAAT - Cartridge-Based Nucleic Acid Amplification Test; ICJ - ileocecal junction

S. no.	Sample for CBNAAT (total N = 30)	No. of patients n/30 (%)	No. of diagnosed definite abdominal TB	Positive CBNAAT report (n = 11)
1	Peritoneal fluid - bilious/serous/purulent	17 (56.66%)	9/17	05/17 (29.41%)
2	Gastric aspirate	10 (33.33%)	4/10	2/10 (20%)
3	Pleural fluid	2 (6.66%)	0/2	0/2 (0%)
4	Endoscopic biopsies Antrum/duodenum/ICJ	3 (10.00%)	1/3	0/3 (0%)
5	Sputum	2 (6.66%)	1/2	1/2 (50%)
6	Lymph nodes peripheral/abdominal	4 (13.33%)	2/4	2/4 (50%)
7	Omental biopsy	3 (10.00%)	1/3	0/3 (0%)
8	Ileal perforation margin	1 (3.33%)	1/1	1/1 (100%)

**Figure 2:**
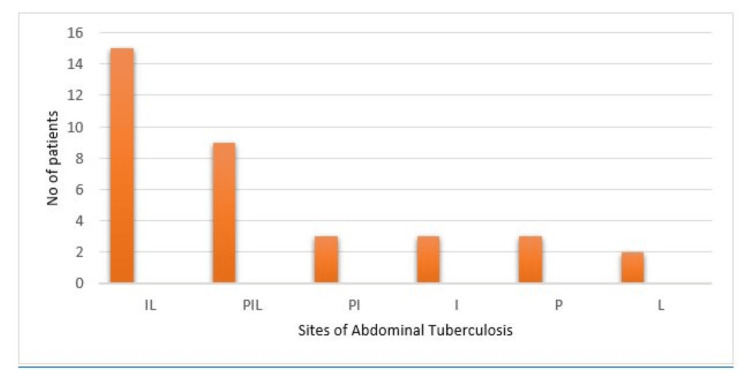
Disease distribution of abdominal tuberculosis in patients I - intestine; L - abdominal lymph nodes; P - peritoneum; V - viscera

Abdominal TB can involve the intestines, abdominal LNs, peritoneum, and visceral organs. In our study population, the most common pattern was concomitant involvement of the intestine and abdominal LNs, observed in 15 out of 34 patients (44.11%). The rest were as follows (Figure 2).

Twenty-six patients (76.47%) underwent CT abdomen, which revealed various suggestive findings of abdominal TB. Four patients underwent endoscopy, the details of which are as follows.

Patient 1

A 13-year-old male who presented with abdominal pain, fever, and altered bowel habits for six months and ultrasound abdomen showed mild hepatomegaly and necrotic abdominal lymphadenopathy underwent upper gastrointestinal endoscopy (UGIE), which was normal, and lower gastrointestinal endoscopy (LGIE), which was superficial, discrete ulcers seen on ICJ and in cecum, where superficial, discrete, transverse ulcers with surrounding erythema were noted. Biopsy revealed chronic inflammation with no granulomatous inflammation.

Patient 2

A 13-year-old male presented with abdominal pain and fever for nine months, loss of appetite and weight for three months, and developed diarrhea with some hematochezia for 15 days. He had circumferential ICJ wall thickening with discrete and conglomerated mesenteric LNs on CECT abdomen. He developed bleeding per rectum in his hospital stay and received a blood transfusion. Colonoscopy revealed an edematous and erythematous ICJ. He had sputum positive for TB and received ATT.

Patient 3

A 16-year-old female presented with abdominal pain, diarrhea, and non-bilious vomiting for two months. Ultrasound and CECT abdomen revealed diffuse circumferential wall thickening of the cecum, ascending colon, ICJ, and distal ileum with multiple LNs. She underwent a colonoscopy, which showed a distorted ileocecal valve and transverse ulcers in the ICV and terminal ileum. Biopsy revealed granulomatous inflammation with negative Ziehl-Neelsen (ZN) staining.

Patient 4

A 14-year-old female presented with chronic diarrhea, loss of appetite, and weight loss. She had wall thickening of the terminal ileum, cecum, and ascending colon, along with moderate ascites on imaging. She underwent a colonoscopy, which revealed edematous mucosa with diffuse ulcers throughout the colon, more in the ascending colon. Biopsy revealed mild chronic inflammation.

**Figure 3:**
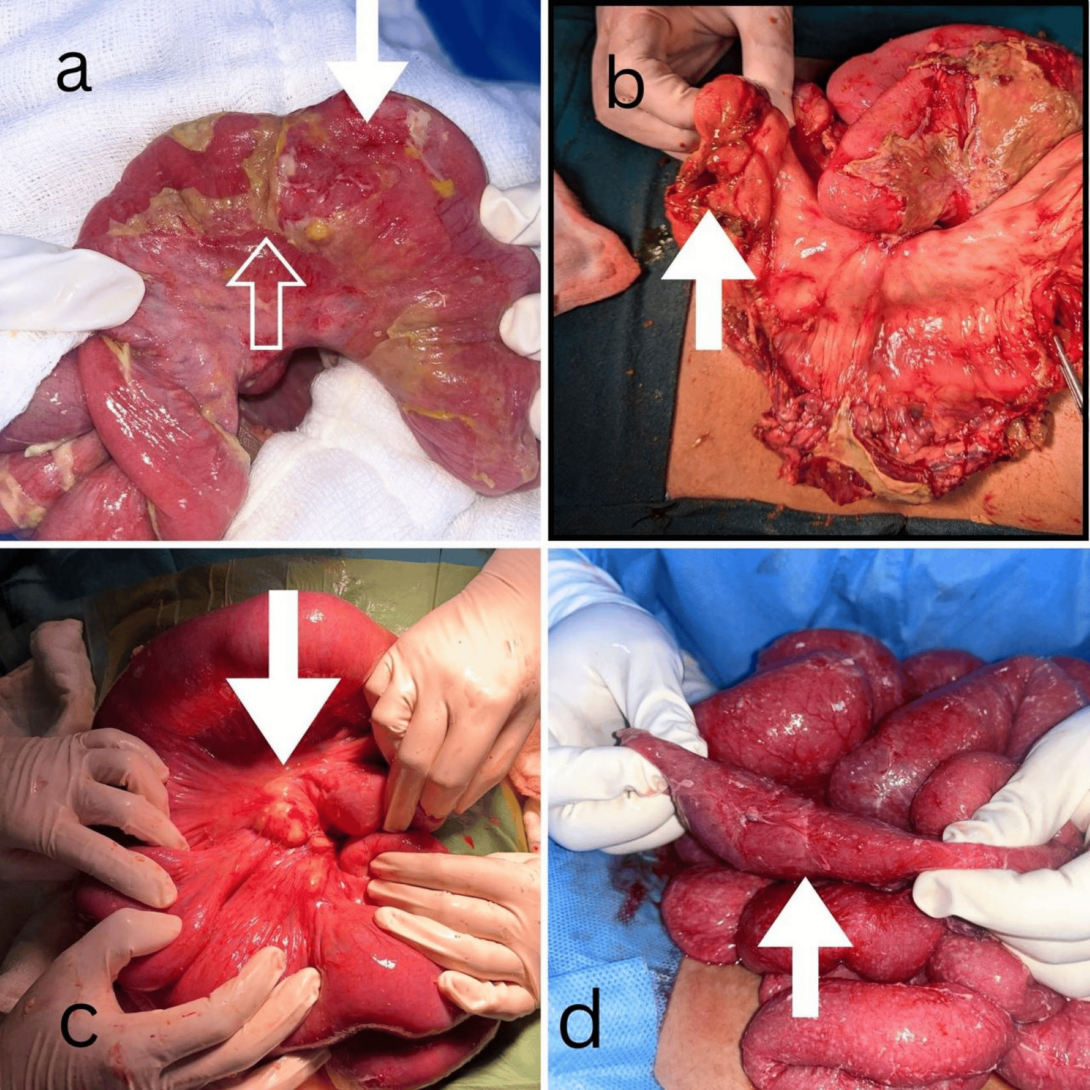
Intraoperative images of the operated cases (a) Passable stricture (white lined arrow) and small perforation (solid white arrow). (b) Large perforation in the ileum (white solid arrow). (c) Caseation of mesenteric lymph nodes (white solid arrow). (d) Passable stricture (white solid arrow) with military deposits over the bowel.

Fifteen patients (44.11%) underwent surgery, 12 for intestinal perforation and three for intestinal obstruction. The distribution in terms of area of bowel perforation was - ileum (5/12), ileum and jejunum (4/12), ileum and caecum area (2/12), and only cecum (1/12). Some of these intestinal perforation cases had associated passable strictures, which were left as such (Figure 3a). Two cases underwent primary repair of the perforations, and the other 10 underwent diversion procedures, especially when the bowel was unhealthy, the perforations were big (Figure 3b), and the children were sick. Two of the obstruction cases had strictures, which were managed with resection and anastomosis of the strictured segments. One of them had a large caseation of the mesenteric LNs (Figure 3c). The third obstruction had features of edematous bowel with ascites, military deposits, and mesenteric lymphadenopathy, and underwent a peritoneal lavage with sampling of the peritoneal fluid and LNs (Figure 3d). A total of seven patients who underwent surgery for intestinal perforation expired. The other four survived and are doing well on follow-up. Two of these cases who survived have undergone a second surgery for restoration of bowel continuity after completion of their ATT medications.

**Figure 4:**
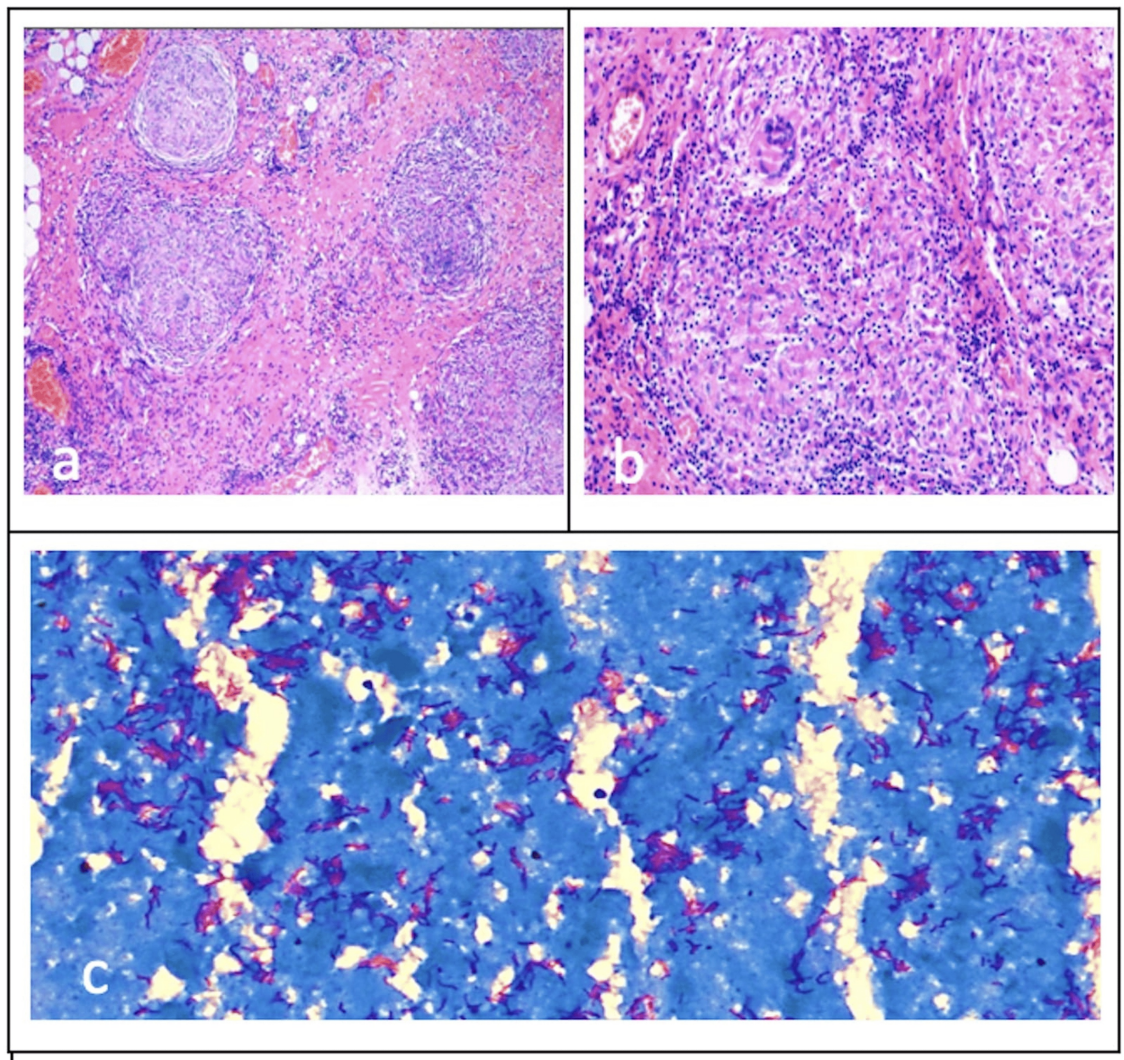
Histopathological images of one patient in the study (a) Omental biopsy shows multiple granulomas creeping into the omental fibroadipose tissue (H&E; ×100). (b) High power shows central caseous necrosis, surrounded by a wall of immune cells, including epithelioid macrophages, multinucleated Langhans giant cells, and lymphocytes (H&E; ×400). (c) Ziehl-Neelsen (ZN) stain demonstrating numerous acid-fast bacilli (AFB) as bright red- or magenta-colored rod-shaped *Mycobacterium tuberculosis* bacteria (ZN; ×1000).

Out of the 18 definite cases of Abdominal Tuberculosis, 11 were CBNAAT positive and 10 had histopathology suggestive of TB (Table 3, Figure 4). The 16 probable cases of Abdominal Tuberculosis had a strong history and imaging suggestive of TB abdomen.

**Table 3:** Details of patient age and gender, with distribution of CBNAAT and histopathology reports CBNAAT - Cartridge-Based Nucleic Acid Amplification Test; LN: lymph node; NGI: necrotizing granulomatous inflammation; ZN: Ziehl-Neelsen stain; (-): sample not available for biopsy

S. no. (patients)	Age (years)	Sex	Diagnosis of TB	CBNAAT	Biopsy (endoscopic/surgical) description
1	15	F	Definite	Negative	Mesenteric LN - necrotizing granulomatous lymphadenitis (NGL); perforation margin - necrotizing granulomatous inflammation (NGI)
2	13	M	Probable	Negative	Antrum, duodenum, and ICJ tissue - nonspecific inflammation
3	6	F	Definite	Positive	Perforation edges: NGI with ZN +
4	12	F	Probable	-	Duodenal biopsy: Chronic nonspecific duodenitis and *Helicobacter pylori*-induced gastritis
5	12	M	Definite	Positive	-
6	10	F	Definite	Positive	-
7	13	M	Definite	Positive	-
8	15	F	Probable	-	-
9	17	F	Definite	-	Perforation edges: NGI with ZN +
10	12	M	Probable	Negative	Cocoon peel tissue: Fibrous peel - granulomatous inflammation
11	16	F	Probable	Negative	ICJ ulcer: Granulomatous inflammation, ZN -
12	12	M	Definite	Positive	-
13	8	F	Definite	Positive	Perforation edge and omentum: NGI, ZN -
14	17	F	Definite	Positive	ICJ and mesenteric LN: NGI, ZN +
15	14	F	Probable	-	-
16	6	F	Probable	Negative	-
17	14	F	Probable	Negative	Duodenum, ICJ, colon biopsy: Mild chronic inflammation of duodenum and gastric, normal colon
18	15	F	Definite	Negative	Resected bowel: NGI
19	13	F	Probable	Negative	-
20	17.5	F	Definite	Negative; indeterminate	Perforation edge: NGI
21	4	F	Definite	Positive	-
22	16	F	Definite	Negative	Ileal polyp, colon: NGI
23	10	F	Probable	Negative	Omental biopsy: Necrotizing non-granulomatous inflammation
24	14	F	Definite	Negative	Mesenteric LN, omentum, and stoma site: NGI
25	15	M	Probable	Negative	Omentum and interbowel bands: Necrotizing inflammation
26	14	M	Probable	Negative	-
27	13	F	Probable	Negative	-
28	5	M	Probable	Negative	-
29	15	F	Definite	Negative	Ileal perforation edge: NGI with ZN -
30	16	F	Definite	Positive	Ileal perforation edge: Acute on chronic inflammation and foci of necrosis with no e/o granuloma or malignancy
31	4	M	Definite	Positive	Periportal LN: NGI
32	11	F	Probable	Negative	-
33	13	F	Definite	Positive	-
34	8	M	Probable	Negative	-

The following table elaborates on the follow-up of the study population (Table 4).

**Table 4:** Follow-up of the study population

Parameter	Findings
Follow-up	Median 13 months (range: 6-30 months)
Treatment completion	All patients completed antitubercular therapy (ATT) (two-month intensive + four-month continuation), except one relapse and one default (later restarted)
Clinical improvement	Achieved in all patients who completed treatment
Complications	Four cases of drug-induced liver injury (DILI) during ATT; managed with temporary second-line therapy until liver function normalized, then first-line resumed
Mortality	Seven deaths, all in patients with perforation peritonitis

## Discussion

Abdominal TB continues to be a common and challenging abdominal disease in children, often requiring admission due to the symptoms, a myriad of investigations before initiation of the prolonged oral therapy, a long duration to diagnose the disease and initiate antitubercular drugs, the low sensitivity and specificity of the tests done to clinch this disease early and finally a poor outcome in cases where the disease progresses to require surgery. The need for aggressive resuscitation and carrying out of necessary available tests from various fluids or tissues from patients to diagnose it early is the need of the hour.

We admitted a total of 47 patients with suspected complaints of abdominal TB, but 13 were diagnosed as having some other abdominal pathology during their hospital stay. Abdominal TB should be differentiated from other diseases presenting with malabsorption (celiac disease, lymphomas); mass per abdomen (lymphomas, Crohn’s disease, appendicular lumps, cecal carcinomas); and ascites (malignancy, diseases of renal, hepatic, or cardiac; pelvic inflammatory disease) [11,12].

The male-to-female ratio in our population was 1:2.4. Mehraj et al., in their review of 31 studies to assess the extent of gender disparity of extrapulmonary TB (EPTB) in a few developing countries, reported that while TB was more prevalent in males, a higher preponderance of EPTB was observed in females [13].

The mean time taken by patients to reach our tertiary center, where diagnosis was made, was 4.32 ± 5.52 months. Twenty-two (64.70%) patients received treatment from other hospitals for their symptoms before presentation to our center. All these cases were newly diagnosed and initiated on ATT at our center during this study duration. This timeline shows the difficulty in diagnosing abdominal TB and the challenges that the family goes through in the process.

The commonest symptoms in our population were abdominal pain (94.11%), fever (73.52%), and loss of appetite and weight (70.58%). The three symptoms were seen in 21/34 patients (61.76%). Lal et al. in their study of 218 cases of abdominal TB in children said that perhaps these three symptoms together could be called the “Triad of Abdominal Tuberculosis” [10].

A significant proportion of our patients were sick on presentation (50%) with dehydration, emaciation, and features of septic shock. Also, 21/34 (61.76%) of the children were underweight, and 35.29% had pallor by the time they presented to us, indicating the nonspecific nature of the disease, making the detection delayed in this part of the country. The total duration of the symptoms that the patients suffered from at the time of presentation to the institute was 4.32 ± 5.52 months. Lal et al. showed a similar median delay of four months from the onset of symptoms to the diagnosis [10]. This may highlight the importance of having a lower threshold to initiate investigations, particularly the diagnostic imagings, which seem to play a crucial role in the early diagnosis of TB, guiding biopsy, evaluating response to treatment, and identifying complications [14].

Although the study was prospectively designed to include routine investigations such as CBNAAT for all patients, samples could be successfully obtained in only 30 cases. The 11 patients who showed CBNAAT positivity were in the following samples: peritoneal fluid, gastric aspirate, sputum, LNs, and ileal perforation margin tissue. In the recently published meta-analysis by the WHO, the sensitivity of CBNAAT in diagnosing various EPTB, such as LN TB, CNS TB, and pleural TB, is 84.9%, 79.5%, and 43.7%, respectively, compared to culture [15]. Nishal et al. [9] analyzed in their study the yield of CBNAAT in extrapulmonary TB and concluded that CBNAAT was positive in 30.76% of EPTB cases. The highest yield was for bone and joint TB (35.7%), followed by LN TB (34%) and abdominal TB (33.3%) [9]. They concluded that, as a single diagnostic tool, HPE had the highest sensitivity in diagnosing extrapulmonary TB. Puri et al. in their analysis of 77 patients of TB abdomen managed surgically got a sensitivity rate of 29% for CBNAAT samples and a sensitivity of 75.3% in Histopathology reports [16]. They also described the cases where CBNAAT can be falsely negative - improper sample, errors in thermolabile cartridges storage, and presumptive ATT intake. 

Nineteen patients were managed with ATT, and the response was good - one child relapsed and needed a further extended course of ATT, four patients developed drug-induced liver injury (DILI) due to ATT and had to be managed with second-line ATT till the liver function normalized, and first-line ATT could be resumed. One patient had resistance to Rifampicin and needed the next line of ATT. The response to ATT was achieved in most cases, though complicated by episodes of hepatotoxicity and the emergence of drug resistance in a few patients. Other studies also show excellent response to ATT in abdominal TB [17].

The role of surgery is usually in the management of complications like obstruction, perforation, bleeding, etc., and less commonly, but worth discussing in the cases of diagnostic uncertainty, wherein laparoscopy and biopsies have had high histological sensitivity from 85 to 100% [18-20]. In India, about 3%-20% of bowel obstructions and 5%-9% of gastrointestinal perforations (next to typhoid) occur due to abdominal TB [20-22]. Undiagnosed and untreated intestinal TB can carry a mortality rate reaching up to 60%, whereas treated abdominal TB carries a mortality rate close to 15% [23-25]. In particular, intestinal TB can lead to perforation, which carries a mortality rate of 30% [25]. The mortality rate amongst the perforations in our population has been 58.33%, highlighting the delay in seeking medical help by the family, referral by the local medical centers, and hence the delay in diagnosis and poor outcome. In contrast, all patients with strictures or those managed medically with ATT survived.

Limitations and strengths

Our study had a relatively small sample size of 34 patients, which is fewer than those included in some other observational studies on abdominal TB. Although the study was initially designed to be prospective, certain investigations could not be performed in all cases. Tests, such as the Mantoux test and fluid ADA levels, were not available for every patient. CBNAAT was conducted in only 30 patients, primarily due to insufficient fluid volume or the unavailability of fluid as samples. Additionally, LN sampling was not feasible in all cases, limiting microbiological confirmation in some patients. A longer duration of follow-up could have provided a more comprehensive understanding of treatment outcomes and disease progression.

It is an initial prospective observational study from an endemic region spanning three years. It includes both medical and surgical cases of pediatric abdominal TB, which is less commonly reported in prior literature. Our study stratifies patients into definite and probable TB based on standardized microbiological and histological definitions.

## Conclusions

TB of the abdomen continues to plague the pediatric population in India. The need for preventive measures in families with TB prevails. There is a dire urgency to seek medical attention and lower the threshold of getting early imaging done in patients having persistence of problems not settled by the initial line of medications. It is essential to emphasize the curability of abdominal TB while simultaneously enhancing education and awareness at both the primary care level and within the community. Seeking help early and prioritizing treatment will be a key factor in decreasing the morbidity and mortality of this challenging, long-standing disease.
